# “In some ways it feels like a specialism”: Exploring the lived experience of multilingual maternity professionals – A qualitative interview study

**DOI:** 10.1016/j.pecinn.2025.100378

**Published:** 2025-01-22

**Authors:** Emma Brooks

**Affiliations:** International Centre for Intercultural Studies, UCL Institute of Education, London, UK

**Keywords:** Multilingualism, Diversity, Patient experience, Workplace equity, EDI

## Abstract

**Objective:**

This study aimed to investigate the experience of multilingual maternity staff working in UK NHS hospitals.

**Methods:**

As part of an exploratory qualitative descriptive approach, semi-structured interviews were conducted with multilingual healthcare professionals, working in perinatal care in different NHS trusts across the United Kingdom. Interviews were audio-recorded, transcribed and subsequently analyzed using thematic analysis.

**Results:**

Where practitioners were able to draw on their linguistic skills, they felt that multilingualism was a specialism and appreciated by colleagues. Practitioners also felt that the utilisation of shared languages could boost the confidence of women and birthing people, as well as improving their understanding and sense of wellbeing. Conversely, several practitioners felt an obligation to offer linguistic support, noting that it added to a workload burden, and fear of litigation, that was not experienced by monolingual colleagues.

**Conclusion:**

Strategic utilisation of linguistically skilled NHS practitioners may hold the potential for advancing equity of care for migrant populations, who are regularly and disproportionately represented in data recording adverse outcomes.

**Innovation:**

Investing in institutional support and formal accreditation for multilingual health professionals would enable them to be able to operate with confidence, redress (invisibilized) workloads and contribute to advancing parity of care for migrant patients.

## Highlights

• Many NHS employees speak additional and heritage languages.
• Language concordant care may enhance patient wellbeing and experience.
• Multilingual medical professionals have communicative skills which need recognition.
• Informal interpreting may hold EDI implications.

## 1 Introduction

From the perspective of established literature focussing on maternity provision in the UK, it is recognised that migrants may encounter difficulties in accessing health services, as well as clinical care that meets their complex needs [[1], [2], [3], [4]]. It is also well-documented that people born outside the UK, those of Black and minority ethnicity and/or with limited English proficiency are disproportionately represented in maternal mortality rates [5]. While discussions surrounding migration and healthcare have frequently focused on language as a barrier to health [[6], [7], [8]], a growing recognition of language as a key social determinant of health can be seen to amplify their salience [9,10]. Professional interpretation and translation are therefore regarded as integral to ensuring effective facilitation, improved comprehension and crucial to equitable care (see for example, [[11], [12], [13]]).

However, to date, a communicative resource that remains largely unacknowledged and underexplored is that of the National Health Service (NHS) workforce itself: for example, no data is currently collated on ‘languages spoken’, despite the fact that approximately 19 % of staff have a nationality other than English, 26 % are of Black or minority ethnicity and one in five individuals in the UK are estimated to speak [2,14] (a) heritage language(s) [14,15]. While figures may yet change in response to recent political moves to curb economic migration [16], this decade has seen a significant change in the number of international healthcare professionals entering the UK workforce: in 2023, almost half of all midwives joining the NHS were educated overseas, whereas 52 % of new medical graduates who registered with the GMC qualified outside the UK or the EEA [16,17]. In combination, and while it is important not to conflate (inter)national or ethnolinguistic characteristics (see for example [18]), data suggests that the NHS workforce is likely to mirror the diversity of the wider patient population.

Emergent evidence that healthcare practitioners draw on their linguistic resources, or those of bilingual colleagues, to communicate more effectively with patients and their families has been noted in a number of contexts (see for example, [[19], [20], [21], [22], [23]]). While expedience may be a factor in emergency circumstances [24], several studies document a more routine dimension, with multilingual encounters taking place in a range of hospital settings. Patriksson et al. [25] demonstrate that the reassurance of a familiar language can put people at ease, consolidate comprehension and can enable them to become actively involved in their own care or those of unwell family members. As bilingual staff have knowledge of technical vocabulary and medical procedures [26], the use of a shared language may also mitigate the potential for misunderstanding and breakdowns in communication, which are recognised challenges of language discordant and/or professionally mediated interaction (see for example, [[27], [28], [29], [30]]). Indeed, in the United States, the apparent benefits of linguistic concordance have led to concerted advocacy for training to support bilingual professionals [31,32].

In a previous study looking specifically at multilingualism within the NHS nursing population, Ali and Johnson [33] find that the choice to conduct consultations in a patient's preferred language is dependent on contextual factors: these include a practitioner's confidence in their own language skills, the expectations of patients and/or how multilingualism is perceived more widely by colleagues, the department or the organization. On the other hand, although their research on language concordant care in the NHS illustrates that it has a demonstrably positive effect on improving patient experience and outcomes, health practitioners voice concerns about the additional workload induced by interpreting, a sense of obligation to meet the expectations of colleagues and patients, and anxieties about accountability (see also [25]).

Nevertheless, with the exception of Ali and Johnson's foundational work, the linguistic diversity of the NHS workforce still remains largely unexplored, either as a topic of enquiry or as an untapped professional resource. This may be because current NHS guidelines on interpreting and translation in primary care [34] advise medical staff to refrain from using additional languages to communicate with patients, ‘other than where immediate and necessary treatment is required’ (2018:8), i.e. emergency situations, or ‘unless this is part of their defined job role and they are qualified to do so’ (ibid). Evidence for guidance seems to be based on a ‘common sense’ [35] approach to language, rather than empirical substantiation. This study therefore aims to contribute to our understanding of the everyday communicative experiences of multilingual NHS healthcare professionals, to explore the extent to which they (feel able to) draw on languages other than English in consultations and to what effect. In conclusion, several recommendations are made, as well as suggestions for further research.

## 2 Methods

Taking a qualitative descriptive design, this study seeks to build on the limited number of studies looking at the multilingual practices of medical practitioners in clinical settings. As Doyle et al. [36] outline, when little is known about a particular topic or lived experience, qualitative descriptive inquiry can help to explore individual subjectivities in order to augment existing knowledge and/or to lay the groundwork for a larger study. This type of approach is sympathetic to an understanding that realities are multiple, dynamic and frequently context bound. Although researchers working within the paradigm do not seek to extrapolate beyond the confines of a study, a qualitative descriptive design can help to highlight areas worthy of further interrogation.

For the purpose of this study, a combination of methods was used to recruit research participants. In the first instance, a flier was disseminated via social media and a professional body who had agreed to advertise recruitment for the project: a multilingual specialist who worked for the organization also took part and subsequently introduced the researcher to other potential participants. Simultaneously, additional interviewees were recruited through a network of practitioners working in maternity care and who had expressed an interest in the study. In total 11 individuals responded and volunteered to participate in their own time: each received a book token by way of compensation. The interview cohort held differing levels of experience and seniority in their roles: it comprised three consultants, six midwives, with two holding roles as educators and one working in policy, a newly qualified midwife and one trainee. Within this group, two had 0–5 years' experience, seven had between 6 and 10 years' experience and two had been working in the profession for more than 15 years. Interviewees participated from different geographical areas across the UK: six worked at London NHS trusts, two worked for hospitals in the Midlands, and the remaining three were located in the North-East and South-East of England, and Scotland. Of the interviewees, 7 were heritage language speakers.

The study was approved by University College London. After receiving information about the research, and how data would be processed and stored securely, all participants gave full written consent. In an effort to guarantee confidentiality, individuals were anonymised during the transcription process, and all data disaggregated, so that no identifiable information could be linked to either participants or institutions. Participants were invited to take part in individual interviews which explored their experiences of using languages other than English during consultations or in the workplace. Interviews took place between February and July 2022: they were conducted via Zoom, in order to mitigate post-pandemic health anxieties, as well as to accommodate the unpredictable nature of interviewees' shift patterns.

Semi-structured interviews offer the opportunity for flexible inquiry, simultaneously providing a systematic framework for the questioning process, yet space for accommodating unanticipated or interesting information and ideas that may emerge during interaction. However, there is a responsibility to acknowledge that interviews are a social practice [37], the dialogic nature of which may inadvertently influence responses because of the nature of the questions posed or the ways in which they are asked [38]. Reflexivity is therefore a central consideration of the qualitative researcher, compelling the need to deliberate on the potential for co-construction and the subsequent interpretation of findings. In a first step towards mitigating a possible influence on content, recorded interviews were transcribed verbatim and returned to participants for member-checking. This stage offered individuals the opportunity to (re)consider their contributions and amend if they wished. Once transcript content was agreed, the researcher followed Braun and Clarke's recommendations for thematic analysis [39]: familiarising themselves with the data, they reviewed content several times and made notes, before beginning to code by hand. Coding involved a combination of induction, where codes were generated from the data, and deduction which brought findings from previous studies to bear on the content. Through a process of iterative review, discrete codes were established and, where codes were very similar, they were collapsed into one another. This continued until overarching themes were identified.

## 3 Findings

Following analysis, six dominant themes were identified: the linguistically diverse workforce; the role of language in patient-centred care; the ways in which language offers a cultural bridge and emotional support for women; an understanding of linguistic skills as a specialism and a source of professional pride; reservations about (a lack of) proficiency; and finally, reflections on the experience of being an (in)valuable, but often invisible, institutional resource. Illustrative quotes are to be found in Table 1 below.

**Table 1:** Interview findings.

A multilingual workforce	*“We tend to collect doctors in little groups in the same way that you collect…patients in little groups, so there are more Italian doctors and Greek doctors here than on average in the NHS in the same way there are more patients who are Greek or Italian.” (P5)*	*“I know what some of the midwives speak and I'll go and find them if I need translations” (P5)*
Patient-centred care	*I think that if they don't speak English, it gives them a better experience of their journey, whatever that is….often feel when people don't speak English… in the healthcare system, they feel very detached from what's happening to them…especially if they don't understand anything. (P11)*	*She didn't feel like she had to change her ways to meet the system. The NHS system altered to meet her needs and I think that's how we should be going forward. (P9)*
Emotional and cultural brokering	*Because I'm coming from outside and they're coming from outside, I'm sort of a bridge between the home medicine in obstetrics, gynaecology and British one - they trust me.” (P8)*	*“So I started to speak to them in Urdu, and honestly you can just see in their face that relief that they can express themselves to somebody without having to think about what to say you know. There was that connection straight away.” (P4)*
	*I think because I was able to speak to her in her language, she instantly felt at ease. And she because she could openly ask me her questions. She didn't think, oh, I'm saying the right thing or the right words because it was natural for her to speak Punjabi. (P9)*	
Professional linguistic pride	*“I was the midwife looking after [a woman], the senior registrar also spoke Cantonese, the midwife coordinator also speaks Cantonese, and she went for a [caesarean] section and the anaesthetist also spoke Cantonese. It was like the universe aligned…it just so happened…the day that you are admitted to hospital everyone on the unit speaks your language…honestly I don't think I'll ever forget that shift, where I thought it's like we all were here for this woman.” (P1)*	*In some ways it feels like… a specialism in terms of what you're able to offer to the people you're working with and that you're caring for…just in terms of trust and relationship building, because often in maternity you know you're meeting someone and all of a sudden you're with them for the most intense, frightening exciting roller coaster.” (P7)*
		*I felt proud that I can help and that's people recognizing me as a potential source of help, and I can be trusted that I can translate what they want me to translate. (P8)*
Linguistic reservations	*I'm good at French. I'm good at maternity. Could I translate you know a liver transplant? Maybe not. There's a limit to how far you could stretch. (P5)*	*I've learned my Punjabi, my Hindi very conversationally with my folks. We don't talk about vaginas… we don't talk about that stuff. I've had to learn ….and so actually being able to do a direct one-to-one translation is quite hard. (P2)*
	*When I was trying to have conversations with colleagues in Italy, like, I had totally blanked out everything that I've learned before and I could only use English words to talk about…..instrumental delivery. (P11)*	*a lot of people are just doing it [interpreting], you know, to be kind to help out, and I think I worry about it coming back to bite them (P5)*
An (in)valuable resource	*“I mean…one of the issues I have…in the antenatal clinic where I work …one of the healthcare assistants speaks Polish well …literally every day she gets called at least twice a day to interpret ….I got so upset - like we are running a service, right? You know she can't be pulled away every time. …they literally they don't even ask me. They just come and get her… also, this is taking it away from the care she's delivering to the antenatal women.” (P11)*	*Sometimes it can be problematic when healthcare professionals are being pulled from people… they're caring for to go and interpret for someone else because it adds to our workload massively…also gives us a sense of responsibility because there's a connection with these people you know (P6)*
		*you don't get an uplift on your salary, because you can default to another language and offer these services ….it is invisible (P10)*

### 3.1 A multilingual workforce

Many of the research participants report working in a linguistically, culturally and ethnically diverse environment. So much so, that several interviewees reported informal departmental initiatives where staff languages were recorded on a local computer database, with internal contact details. In these settings, staff volunteered for the register and could expect to be asked to interpret for patients should it be needed. In one maternity unit, at a large NHS hospital, the unofficial database held a record of over 50 multilingual staff, speaking more than 24 languages collectively. One interviewee noted that the nationality and languages of the staff tended to echo those of the local area.

*“We tend to collect doctors in little groups in the same way that you collect…patients in little groups, so there are more Italian doctors and Greek doctors here than on average in the NHS in the same way there are more patients who are Greek or Italian.” (P5)*

### 3.2 Patient-centred care

Multilingual healthcare workers are sympathetic to the stress of potential language barriers, appreciating that “*when people don't speak English… in the healthcare system, they feel very detached from what's happening to them…especially if they don't understand anything”. (P11).* Almost unanimously, the interviewees described the sense of relief, reassurance and trust that they believe could be gained through the use of a familiar language,

“*So I started to speak to them in Urdu, and honestly you can just see in their face that relief that they can express themselves to somebody without having to think about what to say, you know. There was that connection straight away.” (P4).*

One consultant concludes that language concordant care reflects the “*NHS system alter*[ing] *to meet the needs*” (P2) of individuals, offering the potential of empowerment and giving them “a *better experience of their journey” (P11*). They note, *“I do think being able to speak to women directly in their language really empowers them to make their own decisions …about their own bodily autonomy” (P2).* Reflecting on receptive aspects of interaction, an experienced midwife educator argues that being able to understand what women and birthing people are saying, especially when distressed or in labour, allows the professional to support processes of (un)informed consent.

*“There's been times when I've been able to advocate properly and in an informed way. There's been times, where I've had to stop basically obstetric violence occurring … because they've said something that I've recognized, but no one else has” (P6)*

### 3.3 Emotional and cultural brokering

While linguistic concordant interaction seems central to improving comprehension, experience and outcomes, a familiarity with cultural contexts can also facilitate relationships and ongoing care. A multilingual consultant, who grew up in Eastern Europe, relates a story about advising a pregnant Ukrainian refugee who was presented with a medical decision:

*“When she saw my name … she literally swiftly went to Russian and …that relieved the pressure immediately and the question she asked was like what you would do if you if you've been in* [place]… *what would you advise me and I said exactly the same what I advise you here, okay then I will take it.” (P8).*

The consultant understood the cultural connection to facilitate trust, stating that “*because I'm coming from outside and they're coming from outside, I'm sort of a bridge between the home medicine in obstetrics, gynaecology and British one - they trust me.” (P8).* Other research participants are also keen to emphasise the importance of cultural knowledge: for example, a midwife with a South Asian background stresses the need to contextualise information with diasporic populations, in a way that is both relevant and helpful. They exemplify by relating advice frequently given to women in their care:

*“Gestational diabetes is not the same as regular diabetes… it's not gonna be the same challenges as your uncle and aunts are gonna have… but also I fully appreciate our food, our shared culture in food, and diet and exercise makes this hard and we need to … make the pregnancy safe for you” (P2)*

### 3.4 Professional and linguistic pride

Participants overwhelmingly see their linguistic skills as a specialism, that enables them to support women and birthing people on a physically and emotionally demanding journey.

*“In some ways it feels like…a specialism in terms of what you're able to offer to the people you're working with and that you're caring for…just in terms of trust and relationship building, because often in maternity you know you're meeting someone and all of a sudden, you're with them for the most intense, frightening exciting roller coaster.” (P7).*

While some multilingual professionals are the sole speakers of a specific language in their team, others work in trusts where they share repertoires with colleagues. A newly qualified midwife relates her surprise at finding herself working with other Cantonese speakers at the time of an emergency admission.

*“I was the midwife looking after [a woman], the senior registrar also spoke Cantonese, the midwife coordinator also speaks Cantonese, and she went for a [caesarean] section and the anaesthetist also spoke Cantonese. It was like the universe aligned…it just so happened…the day that you are admitted to hospital everyone on the unit speaks your language…honestly I don't think I'll ever forget that shift, where I thought it's like we all were here for this woman.” (P1).*

Similarly, all interviewees express a sense of personal pride in being able to support people linguistically in an unfamiliar and possibly frightening environment. They derive a great deal of satisfaction and happiness from helping workplace colleagues and are proud that their skills are valued, and they are trusted to interpret: *“It felt like I was valued as a team member, and they valued my diversity and that they see me as an asset to the team.” (P9)*

### 3.5 Linguistic reservations

In the workplace, research participants had received varied, and sometimes ambiguous, advice on interpreting and translation, and this prompts a mixture of responses. While some practitioners assume that multilingual midwives should interpret whenever it is needed, others believe that they need a qualification to practice in (an)other language(s). A lack of clarity leads to unease about potential litigation, should interpreting later by identified as a factor in a serious incident investigation. A senior consultant (P5), who is certified to practice in French as well as English, voices concerns on behalf of their colleagues, “*a lot of people are just doing it [interpreting], you know, to be kind to help out, and I think I worry about it coming back to bite them”.* Yet they also concede the limitations of working outside their own specialism: “*I'm good at French. I'm good at maternity. Could I translate, you know, a liver transplant? Maybe not. There's a limit to how far you could stretch”.*

In contrast, midwives who speak heritage languages, i.e. minority languages spoken by parents and learned as children in the home environment, have alternative concerns about the use of technical terms. Anxious about the possible informality of their repertoire used in the home, and potential gaps in their knowledge, one interviewee teaches herself medical terminology in other languages in order to ensure accuracy.

*“I've learned my Punjabi, my Hindi, very conversationally with my folks. We don't talk about vaginas… we don't talk about that stuff. I've had to learn…and so actually being able to do a direct one-to-one translation is quite hard.” (P2)*

### 3.6 An (in)valuable resource

The majority of maternity professionals interviewed are very positive about the advantages presented by their flexible repertoire. While many interviewees recognise the added responsibility, they nevertheless feel valued: they also understood why asking a Spanish midwife to communicate with a Spanish speaking woman, for example, would be preferable to employing a professional interpreter:

*“[It] is about utilizing your resources in isn't it yeah, you've got a skill set within the team, so of course you're going to use that rather than pay for interpreting services –* [a professional interpreter is] *not going to be as good…as a Spanish speaking midwife.” (P10).*

However, some multilingual healthcare staff feel that there is an unspoken expectation among their colleagues that they will routinely help as interpreters, even if it means that they are taken away from someone who is benefiting from their specialist clinical support. In these situations, they feel that language concordance seems to supersede medical expertise, despite the option and availability of professional language service providers. Multilingual practitioners note that it is also difficult to disappoint peers, although the uncompensated work may give them an increased sense of frustration and exploitation.

*“Sometimes it can be problematic when healthcare professionals are being pulled from people they're caring for, to go and interpret for someone else because it adds to our workload massively…also gives us a sense of responsibility because there's a connection with these people.” (P6).*

Several managerial staff express similar dissatisfaction with the uneven workload burden, as they are unhappy with the knock-on effects of multilingual colleagues being asked to adopt different roles simultaneously.

*“I mean…one of the issues I have ..in the antenatal clinic where I work …one of the healthcare assistants speaks Polish well …literally every day she gets called at least twice a day to interpret …I got so upset - like we are running a service, right? You know she can't be pulled away every time…they literally… don't even ask me. They just come and get her…also, this is taking it away from the care she's delivering to the antenatal women.”*

*(P11)*

## 4 Discussion and conclusion

### 4.1 Discussion

This study illustrates some of the everyday complexities of navigating and managing linguistic diversity in the NHS. It contributes to a body of research that shows both the interactional and cultural sensitivity of maternity and nursing practitioners [40,41], as well as their ability to be linguistically resourceful [33,42]. Consistent with work from earlier studies [25,33], participant multilinguals with a range of maternity experience and from different geographical areas in the UK, appeared unanimous in their belief that language concordant care could bring a wealth of benefits for migrant patients, who speak languages other than English. Medical practitioners interviewed asserted that concordance appears to improve patient wellbeing and boost their confidence to ask questions and check comprehension. Patients are not only afforded increased autonomy, but staff with whom they share languages are also in a position to advocate or intervene, should this be necessary. Although it was not mentioned by interviewees, an additional benefit of multilingual staff may be the expedience of support, and the pressure it can take off the already over-stretched interpreting providers, who often struggle to meet the demand on services [7]. Findings suggest that multilingual healthcare professionals take pride in their linguistic skills and feel valued by service users and colleagues alike. A midwife interviewed offered an insight into the effects that a shared language can bring: they recalled a woman returning to the maternity ward two years after giving birth, to ask questions about her toddler's health. When asked why she had travelled across London to do so, and to a non-specialist, the woman responded that the midwife was the only person she had been able to understand and trust.

However, staff interpreting was not necessarily regarded as unproblematic, and it may also be advantageous to investigate the phenomenon in relation to proficiency, patient safety, and workplace equity. In line with a previous study exploring the experiences of multilingual NHS nurses [33], an ambiguity around guidance for the use of shared languages was a reoccurring theme, with several practitioners expressing anxieties about accountability and support. Indeed, there is no doubt that with increased responsibility comes the potential for increased risk and liability and this is an aspect of ad hoc interpretation that would benefit from closer scrutiny. For example, should an adverse event occur, where patient safety is jeopardised, it is not clear whether professional liability would rest with a hospital trust or the healthcare professional. Some staff also related feeling under duress to meet interpreting obligations on top of their regular workload. Without explicit guidelines, institutional recognition of additional competencies, adjusted shifts or any form of compensation, they felt that linguistic labour was invisibilised. If unofficial interpreting adds to a workload that is not experienced by monolingual counterparts, it seems that language may be an aspect of equality, diversity and inclusion (EDI) in the workplace that appears to go unrecognised.

Limitations of this research are nevertheless acknowledged: the number of participants is small and self-selecting and therefore findings cannot be considered to represent the experiences of all multilingual healthcare professionals working in the NHS. Similarly, while interviewees offer highly articulate and predominantly positive accounts of using their linguistic skills in the workplace, it must be noted that attitudes to language(s), varieties and dialects may differ considerably, across different geographical and local contexts: this includes institutions, departments, and individuals. With this in mind, this study has sown a seed for future enquiry: in the first instance, it is hoped that a national survey across NHS trusts will shed some light on the scale and situated nature of staff interpretation practices. Further qualitative, ethnographic research will offer the opportunity to explore the thematic strands identified in more depth, and to investigate language as a central constituent of health and workplace (in)equalities. Simultaneous to this, and in a step towards recognition of equality, diversity and inclusion (EDI) commitments to NHS staff, as well as patient-centred care, another emergent recommendation that arises from this study, would be to pursue assessment, accreditation, and compensation for multilingual staff. Greater institutional acknowledgement, support and appropriately adjusted working conditions could significantly enhance their confidence and ability to provide language concordant care, which in turn could advantage local patient populations.

### 4.2 Innovation

This study contributes to an emergent area of research exploring the benefits of linguistically and culturally concordant care for patients with limited proficiency in English. It illustrates how the unique communicative skillset of multilingual medical professionals can hold advantages for diasporic communities, in terms of cultural sensitivity, comprehension and potential outcomes. However, although findings illuminate the strategic and innovative ways in which participants and their colleagues navigate the complexities of a linguistically diverse landscape, participants' experience highlight tensions between policy and practice that are worthy of further exploration.

### 4.3 Conclusion

This research draws attention to the everyday experiences of linguistically skilled professionals working in the NHS. It highlights the fact that the utilisation of shared languages has the potential to enhance patient wellbeing and comprehension, as well as improving the engagement and health of migrant populations.

## Funding

This research was supported by an Impact Grant from UCL Department for Culture, Communication and Media (2021−2022). Findings were initially presented as part of the EALTHY Professional Development webinar series.

## CRediT authorship contribution statement

**Emma Brooks:** Writing – original draft, Supervision, Resources, Project administration, Methodology, Investigation, Funding acquisition, Formal analysis, Data curation, Conceptualization.

## Declaration of competing interest

The author – Emma Brooks - declares that they have no known competing financial interests or personal relationships that could have appeared to influence the work reported in this paper.
